# Thoracic Aortic Dissection in a Patient With Classical Homocystinuria: Implications for Aortic Surveillance

**DOI:** 10.1002/jmd2.70107

**Published:** 2026-07-19

**Authors:** Marisa Chard, Jasmin Simi Zhang, Lesley Turner

**Affiliations:** ^1^ Faculty of Medicine Memorial University St. John's Newfoundland and Labrador Canada

**Keywords:** aortic dissection, aortopathy, classical homocystinuria, cystathionine beta‐synthase deficiency

## Abstract

Classical homocystinuria (OMIM #236300), a rare inherited metabolic disorder caused by cystathionine beta‐synthase (CBS) deficiency, is characterized by markedly elevated homocysteine levels and associated multisystem complications. While the role of homocystinuria in venous thromboembolism is well recognized, there is limited evidence of impact on aortic pathology. We report the first known case of thoracic aortic dissection in a patient with poorly controlled classical homocystinuria. The patient, a 29‐year‐old female, was diagnosed in childhood after presenting with Marfanoid features and neurodevelopmental issues, and had persistently elevated homocysteine levels. She had morbid obesity (BMI: 52.7 kg/m^2^) with no known hypertension or aortic disease. She died suddenly from a ruptured thoracic aortic dissection. Post‐mortem whole exome sequencing confirmed homozygosity for a pathogenic *CBS* mutation NM_000071.2: c.1058C>T; p.(Thr353Met) without any additional variants implicated in monogenic aortopathy or aortic dissection. Notably, her younger brother also has classical homocystinuria but no evidence of aortic dilation to date. This case adds to emerging evidence that elevated homocysteine may contribute to structural vascular damage. Our findings underscore the need to consider aortic surveillance in patients with classical homocystinuria, particularly those with inadequate metabolic control or additional risk factors such as obesity, and highlight the importance of early, sustained treatment to mitigate potential vascular risk.

## Key Points

- This is the first reported case of a thoracic aortic dissection occurring in a patient with classical homocystinuria.
- Long‐term, sustained exposure to severely elevated plasma homocysteine levels is hypothesized to promote extracellular matrix degradation, chronic elastolysis, and smooth muscle cell proliferation, thereby reducing overall aortic wall resilience.
- Regular aortic surveillance via transthoracic echocardiography should be considered for individuals with classical homocystinuria, particularly those with inadequate metabolic control or additional synergistic risk factors such as obesity.

## 1 Introduction

Classical homocystinuria (OMIM #236200) is an autosomal recessive metabolic disorder caused by pathogenic mutations in the *CBS* gene, resulting in deficiency of cystathionine beta‐synthase (CBS) enzymatic activity. This rare condition has a worldwide incidence of approximately 1:344000, but the incidence can vary significantly based on founder mutations; for example, the incidence in Ireland is around 1:65000 [1]. Based on clinical observations at our center, a *CBS* founder mutation, NM_000071.2: c.1058C>T, p.(Thr353Met) (assembly GRCh37/hg19), is suspected in Newfoundland, an island in the easternmost province of Canada, where several patients with classical homocystinuria have been identified.

### 1.1 Metabolism of Homocysteine in Classical Homocystinuria

**FIGURE 1:**
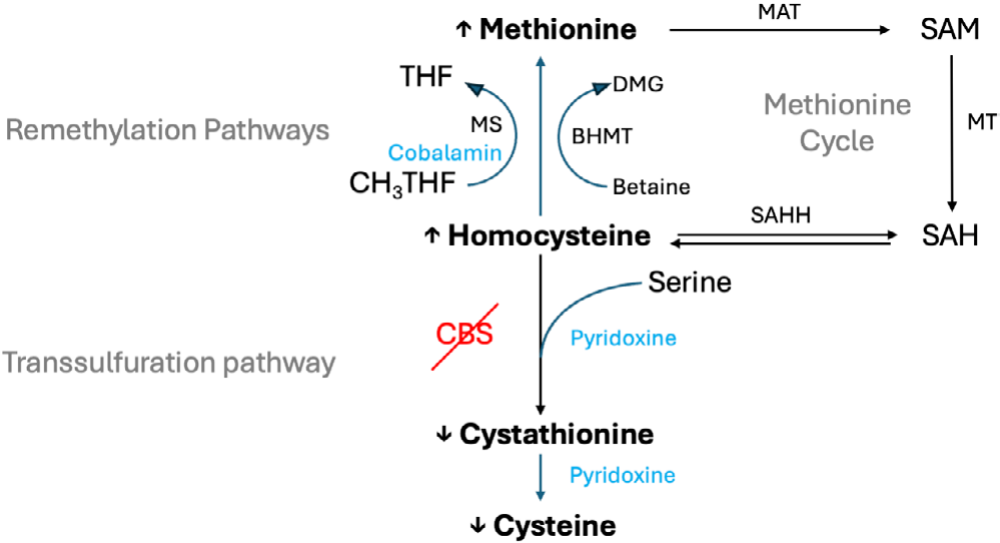
Simplified biochemical pathway involved in classical homocystinuria. Cystathionine *β*‐synthase (CBS) deficiency leads to a disruption of the transsulfuration pathway, resulting in the accumulation of homocysteine and methionine [2]. THF, Tetrahydrofolate; CH_3_THF, 5‐methyltetrahydrofolate; MS, Methionine synthase; BHMT, Betaine‐homocysteine methyltransferase; DMG, Dimethylglycine; MAT, Methionine adenosyltransferase; MT, Methyltransferase; SAHH, S‐adenosylhomocysteinase hydrolase; SAM, S‐adenosyl methionine; SAH, S‐adenosyl homocysteine.

The classical form of homocystinuria arises from a deficiency of CBS, an enzyme responsible for condensing homocysteine and serine into cystathionine in the transsulfuration pathway (Figure 1) [3]. CBS requires pyridoxal‐5′‐phosphate (PLP), and pyridoxine (vitamin B6) is a dietary precursor to PLP. The disorder can be classified as pyridoxine‐responsive or ‐nonresponsive homocystinuria, with the latter being more severe [2]. CBS deficiency leads to an accumulation of homocysteine and methionine, while cysteine levels decrease [2]. Alternatively to transsulfuration, homocysteine can undergo remethylation via methionine synthase or betaine‐homocysteine methyltransferase to regenerate methionine, a pathway that requires folate and cobalamin as cofactors [4]. Betaine can serve as a methyl donor in the remethylation reaction and can significantly reduce homocysteine in B6‐nonresponsive homocystinuria and to near‐normal levels in B6‐responsive homocystinuria [5, 6]. A low methionine diet, betaine, and pyridoxine supplementation are recommended treatments which are tailored to optimize metabolic control in patients with classical homocystinuria [7].

### 1.2 Similarities to Marfan Syndrome

Patients with classical homocystinuria often exhibit physical features similar to Marfan syndrome including a “Marfanoid” body habitus with tall stature, pectus abnormalities, and scoliosis, as well as ectopia lentis. Conversely, vascular thromboembolism and intellectual disability can be features of classical homocystinuria but are not typical features of Marfan syndrome. Given the similarities in clinical features (Table 1), it has been theorized for many years that the two conditions could have similarities in their underlying pathophysiologies [8].

**TABLE 1:** Clinical features of Marfan syndrome and Homocystinuria.

Feature	Marfan syndrome	Homocystinuria
Gene	*FBN1* (autosomal dominant)	*CBS* (autosomal recessive)
Cardiovascular risks	Aortic root dilation/aneurysm/dissection	Thromboembolism, variable aortic involvement
Skeletal features	Tall stature, arachnodactyly, scoliosis, pectus deformities	Similar Marfanoid features but more pronounced osteoporosis
Ocular abnormalities	Ectopia lentis (upward and outward dislocation)	Ectopia lentis (downward and inward dislocation)
Neurological risks	Dural ectasia, normal cognition	Intellectual disability, psychiatric disorders
Homocysteine levels	Normal	Elevated

Patients with Marfan syndrome are known to be at risk for aortic root dilation and aortic dissection [9]; however, there is limited evidence for this in patients with classical homocystinuria and it is largely unknown if patients may be at risk. Guidelines for the diagnosis and management of classical homocystinuria were published in 2017 [7] and they contain no recommendations regarding screening for aortopathy.

## 2 Case Report

Presented here is a patient with poorly controlled classical homocystinuria and morbid obesity who died at age 29 from a thoracic aortic dissection. Clinical histories, exams, and investigations were reviewed. A limited autopsy was conducted, and molecular testing was performed following her death.

### 2.1 Clinical Presentation and Diagnosis

The patient was diagnosed with classical homocystinuria at age of 6 after presenting with speech delay, behavioral issues, myopia, lens subluxation, and Marfanoid body habitus (previously reported [10]). At the time of diagnosis, total homocysteine was elevated at 295 μmol/L and methionine was elevated at 586 μmol/L. She was found to have CBS enzyme deficiency on cultured fibroblasts. She was determined to be pyridoxine unresponsive. A low methionine diet was initiated; however, dietary compliance was poor. Betaine was also started.

### 2.2 Clinical Course and Management

Throughout her life, adherence to treatment had been a challenge. Her plasma total homocysteine levels had been mostly elevated above the recommended target of 100 μmol/L, especially in recent years (Table 2). She was followed by cardiology until age of 18, which appears to have been due to initially suspected Marfan syndrome. Her aorta and aortic root were normal on echocardiogram at age of 17, and imaging had not been repeated since. In adulthood she had developed morbid obesity (BMI: 52.7 kg/m^2^) and psychiatric illness. Due to her mental health issues, she lived in a group home. Adherence to treatment and monitoring worsened as she got older; specifically, there is a significant gap with no total homocysteine measurements between ages 19 and 22. Thereafter, there were no further total homocysteine levels completed up to her death at age of 29. She was not known to have hypertension, and her blood pressure was normal at least 3 years prior to her death.

**TABLE 2:** Patient's average total homocysteine level by age.

Age of patient	Average plasma total homocysteine (μmol/L)	Average plasma methionine (μmol/L)	Number of measurements
7	234.8	807.1	12
8	175.9	912.2	13
9	205.6	937.5	11
10	171.9	797.3	11
11	154.3	796.5	11
12	147.8	709.4	13
13	130.7	753.3	9
14	118.4	739.1	12
15	158.5	903.1	12
16	125.8	855.6	10
17	219.5	971.3	8
18	191.4	766.5	11
19	279.4	847.5	7
22	235.0	672.0	1

### 2.3 Post‐Mortem Findings

**FIGURE 2:**
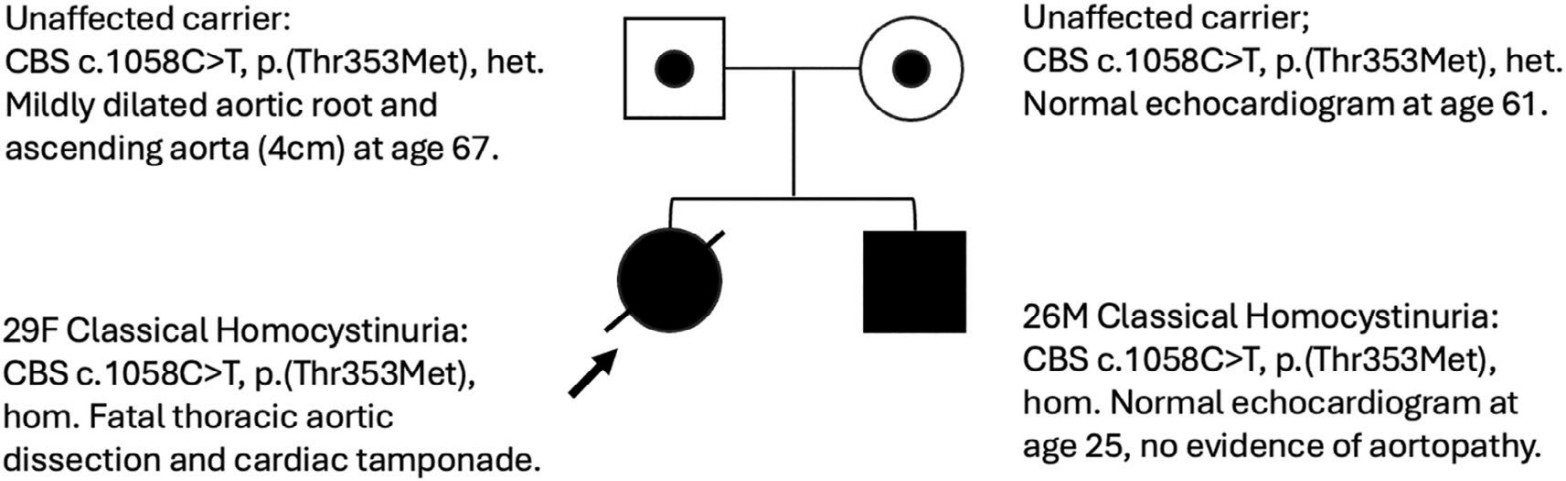
Pedigree: Classical homocystinuria and aortopathy. The proband is indicated by an arrow. Filled symbols denote affected individuals. Hom: Homozygous; het: Heterozygous.

At 29 years old she died suddenly at home. A limited autopsy showed the cause of death to be a ruptured thoracic aortic dissection with cardiac tamponade. Following her death, a genetic panel for aortic disease was performed, which was normal aside from homozygosity for the *CBS* variant NM_000071.2: c.1058C>T, p.(Thr353Met) (assembly GRCh37/hg19). This variant is classified as pathogenic and has been previously identified in patients with classical homocystinuria in combination with another CBS variant [11, 12, 13, 14, 15]. Subsequent trio whole exome sequencing using DNA samples from the patient and her parents did not identify any additional findings, and her parents were confirmed to be carriers of her *CBS* variant (Figure 2).

Following the patient's revealed cause of death, her first‐degree relatives were recommended to have thoracic aorta screening. Her younger brother who is now 26 years old also has poorly controlled classical homocystinuria but has no obesity (BMI: 26) or diagnosed hypertension with normal blood pressure measurements at rest; he was diagnosed shortly after birth due to his sister's diagnosis and for the first 10 years of life his homocysteine levels were mostly below the target of 100 μmol/L. He continues to be followed regularly in the metabolic clinic; however, due to ongoing challenges with treatment adherence, his monthly total homocysteine levels remain persistently elevated, frequently exceeding 200 μmol/L. He had a normal echocardiogram with no evidence of aortopathy, most recently at age 25. Her mother's echocardiogram performed at age 61 was normal. Her father, who has a history of hypertension and hypercholesterolemia, had a screening echocardiogram at age 67 showing a mildly dilated aortic root and ascending aorta. A follow‐up CT angiogram showed some cardiomegaly and the ascending aorta measuring 4 cm; views of the aortic annulus were suboptimal.

## 3 Discussion

To our knowledge, this is the first reported aortic dissection in a patient with classical homocystinuria. The patient did not have any other identified monogenetic cause for the aortic dissection. She was much younger than the age at which idiopathic thoracic aortic dissection is typically seen (usually age 60 to 70) and she did not have known risk factors such as hypertension; however, her morbid obesity likely increased overall cardiovascular and aortic wall stress and contributed to her presentation. Patients with higher BMI have been shown to present with acute aortic dissection at a younger age [16], but reasons for this association have not been fully elucidated. There may be contributions of additional risk factors such as unrecognized hypertensive burden in these patients, and more research is needed to determine the role of obesity as an independent risk factor for aortic dissection.

While the pathophysiology regarding the physical manifestations of classical homocystinuria has not been elucidated, it is speculated that there may be underlying similarities to Marfan syndrome. Marfan syndrome is caused by mutations in the *FBN1* gene resulting in a defective fibrillin‐1 protein. It has been shown that homocysteine reacts with cysteine residues in fibrillin‐1 in vitro disrupting disulfide bonds essential for microfibril assembly [17, 18]. In Marfan syndrome, it is thought that defective fibrillin‐1 contributes to an abnormal extracellular matrix, which may lead to increased activity of TGF‐β, which itself seems to contribute to aortic aneurysm pathogenesis [19]. While a similar mechanism of action is possible for classical homocystinuria, this has not been established.

Several case reports and retrospective studies have highlighted the wide spectrum of vascular abnormalities in patients with classical homocystinuria, including internal carotid artery dissection [20], coronary artery dissection [21], aortic root ectasia [22], abdominal aortic aneurysms [23, 24], and arteriopathy mimicking fibromuscular dysplasia [25]. A case series of 34 patients with classical homocystinuria showed that 7 patients (21%) had some degree of dilation of the aortic root [26]. Notably, this dilation occurred in the absence of ascending aorta involvement or any left ventricular abnormalities, though it was sometimes accompanied by a borderline enlarged ascending aorta and valvular dysfunction, such as aortic or mitral regurgitation [26]. As previously discussed, this pattern of isolated aortic root dilation mirrors the aortopathy seen in Marfan syndrome and may reflect a shared pathophysiological mechanism involving fibrillin‐1 and TGF‐β dysregulation [17]. These findings suggest that aortic root dilation may represent a specific vascular manifestation of classical homocystinuria, even in the absence of a clear dose‐dependent relationship between cumulative homocysteine exposure and aortic root size [26].

Elevated plasma homocysteine levels, even in conditions other than classical homocystinuria, are well documented to be a risk factor for abdominal aortic aneurysm [24], suggesting a biochemical role of homocysteine in aortopathy. High levels of homocysteine exert toxic effects on vascular endothelial cells through several mechanisms. Homocysteine promotes elastin degradation by upregulating matrix metalloproteinases (MMPs) and by forming irreversible bonds with elastin‐dependent proteins, such as fibrillin‐1, thereby compromising vascular integrity [27], mirroring the structural pathology observed in Marfan syndrome. This mechanism is supported by a study of aortic tissue from patients with abdominal aortic aneurysms, which revealed significantly elevated homocysteine levels in aortic smooth muscle cells [23]. Moreover, incubation of aneurysmal tissue with homocysteine led to heightened MMP‐2 activity [28], suggesting a direct role for homocysteine in accelerating extracellular matrix breakdown and predisposing the vessel wall to rupture or dissection. Furthermore, high homocysteine levels enhance the expression of tissue inhibitor of metalloproteinases‐1 (TIMP‐1), which induces an imbalance between MMPs and TIMPs, promoting inflammation and matrix degradation [28]. Homocysteine also induces smooth muscle cell proliferation in the tunica media, or the medial layer of arterial walls, contributing to the loss of vascular elasticity, a key step in the pathogenesis of atherosclerotic plaque formation [29]. Further, homocysteine increases the generation of reactive oxygen species (ROS), leading to oxidative stress and endothelial injury downstream [30, 31, 32]. In our patient, the sustained exposure to total plasma homocysteine levels often exceeding 200 μmol/L likely facilitated a state of chronic, uninhibited elastolysis. While the mechanical stress of morbid obesity likely served as the precipitating trigger, the biochemical groundwork was laid by years of MMP‐mediated medial degeneration, leading to the fatal dissection observed at the relatively young age of 29 [28].

Collectively, these findings illustrate the multifaceted role of homocysteine in vascular pathology, supporting the hypothesis that its accumulation in classical homocystinuria may contribute to the development of aortopathy, including aortic aneurysm and dissection. In the present case, in which total plasma homocysteine levels were often greater than 200 μmol/L across many years, this chronic proteolytic and elastolytic stress may have progressively reduced aortic wall resilience and thereby lowered the threshold for acute rupture or dissection. Although one cannot determine from a single case whether strict metabolic control would have prevented aortic dissection, earlier sustained lowering of homocysteine through diet, betaine, and pyridoxine (if pyridoxine‐responsive subtype) would be expected to reduce long‐term vascular exposure to homocysteine and these potentially toxic effects. In this patient, pyridoxine nonresponsiveness, chronic poor metabolic control, and morbid obesity likely acted synergistically to amplify the overall vascular risk.

The patient's mother and brother (who also has poorly controlled classical homocystinuria) were not found to have any aortopathy. The clinical divergence between the proband and her brother suggests that while biochemical vulnerability is a constant, early diagnosis and stringent metabolic control during critical developmental periods, combined with the maintenance of a normal weight, may have a dramatic impact on preventing the aortopathy observed in this case. Her father was found to have a mildly dilated ascending aorta and cardiomegaly in his late 60s. While her father had environmental risk factors for ascending aortic dilation, including advancing age and hypertension, it may be possible that there were other hereditary risk factors which may have contributed to the patient's aortic dissection.

## 4 Conclusions

Given the reports in the literature showing various types of aortopathy or arteriopathy in patients with classical homocystinuria (CBS deficiency), along with the patient reported herein dying suddenly from an aortic dissection at a young age, consideration should be given to aortic surveillance in patients with classical homocystinuria, especially those with poor metabolic control and/or additional risk factors for aortopathy. Pending disease‐specific evidence, a pragmatic approach would be to obtain baseline transthoracic echocardiography in the late teens or early adulthood, with repeat imaging every 2–3 years in patients who remain metabolically well controlled and more frequent surveillance (e.g., annually) in those who are pyridoxine nonresponsive, have persistently elevated total plasma homocysteine levels, or have additional risk factors such as obesity, hypertension, or a family history of aortic disease. If echocardiographic windows are limited or any aortic dilation is detected, cross‐sectional imaging with MRI or CT should be considered to assess the entire thoracic aorta, consistent with broader aortic disease imaging principles. Surveillance can reasonably be combined with routine cardiovascular follow‐up, including blood pressure assessment and evaluation for associated valvular abnormalities. More research is needed to investigate if there may be a causal relationship between classical homocystinuria and aortopathy.

## Author Contributions

**Marisa Chard:** conceptualization, writing – original draft, review and editing. **Jasmin Simi Zhang:** writing – original draft, review and editing. **Lesley Turner:** writing – review and editing. All authors have read and agreed to the published version of the manuscript.

## Funding

The authors have nothing to report.

## Ethics Statement

Ethics approval was not required for this case report.

## Consent

The proband's parents provided consent for their and their daughter's clinical information to be used in this report. Also, the proband's brother provided consent for his clinical information to be used in this report.

## Conflicts of Interest

The authors declare no conflicts of interest.

## Data Availability

The data that support the findings of this study are available on request from the corresponding author. The data are not publicly available due to privacy or ethical restrictions.
